# Thymoquinone overcomes chemoresistance and enhances the anticancer effects of bortezomib through abrogation of NF-κB regulated gene products in multiple myeloma xenograft mouse model

**DOI:** 10.18632/oncotarget.1596

**Published:** 2013-12-18

**Authors:** Kodappully Sivaraman Siveen, Nurulhuda Mustafa, Feng Li, Radhamani Kannaiyan, Kwang Seok Ahn, Alan Prem Kumar, Wee-Joo Chng, Gautam Sethi1

**Affiliations:** ^1^ Department of Pharmacology, Yong Loo Lin School of Medicine, National University of Singapore, Singapore; ^2^ Cancer Science Institute of Singapore, Centre for Translational Medicine, Singapore; ^3^ Department of Haematology-Oncology, National University Cancer Institute, Singapore, National University Health System, Singapore; ^4^ College of Korean Medicine, Kyung Hee University, Seoul, Republic of Korea; ^5^ Biomedical Sciences, Faculty of Health Sciences, Curtin University, Western Australia, Australia; ^6^ Department of Biological Sciences, University of North Texas, Denton, Texas, USA

**Keywords:** Thymoquinone, MM, bortezomib, apoptosis, NF-κB

## Abstract

Multiple myeloma (MM) is a B cell malignancy characterized by clonal proliferation of plasma cells in the bone marrow. With the advent of novel targeted agents, the median survival rate has increased to 5−7 years. However, majority of patients with myeloma suffer relapse or develop chemoresistance to existing therapeutic agents. Thus, there is a need to develop novel alternative therapies for the treatment of MM. Thus in the present study, we investigated whether thymoquinone (TQ), a bioactive constituent of black seed oil, could suppress the proliferation and induce chemosensitization in human myeloma cells and xenograft mouse model. Our results show that TQ inhibited the proliferation of MM cells irrespective of their sensitivity to doxorubicin, melphalan or bortezomib. Interestingly, TQ treatment also resulted in a significant inhibition in the proliferation of CD138+ cells isolated from MM patient samples in a concentration dependent manner. TQ also potentiated the apoptotic effects of bortezomib in various MM cell lines through the activation of caspase-3, resulting in the cleavage of PARP. TQ treatment also inhibited chemotaxis and invasion induced by CXCL12 in MM cells. Furthermore, in a xenograft mouse model, TQ potentiated the antitumor effects of bortezomib (p < 0.05, vehicle versus bortezomib + TQ; p < 0.05, bortezomib versus bortezomib + TQ), and this correlated with modulation of various markers for survival and angiogenesis, such as Ki-67, vascular endothelial growth factor (VEGF), Bcl-2 and p65 expression. Overall, our results demonstrate that TQ can enhance the anticancer activity of bortezomib *in vitro* and in vivo and may have a substantial potential in the treatment of MM.

## INTRODUCTION

Cancer is a major public health problem worldwide. According to World Cancer Research Fund International, there were an estimated 12.7 million cancer deaths (13% of all deaths) worldwide in 2008, males accounting for 6.6 million and females accounting for 6 million. Multiple myeloma (MM) is a B cell malignancy involving the post germinal center B cells. The disease is characterized by clonal proliferation and accumulation of terminally differentiated plasma cells that produce immunoglobulin [1], presence of blood and urinary monoclonal proteins, osteolytic bone lesions, infiltration of bone marrow with malignant plasma cells [2]. Generally, MM is preceded by two premalignant conditions namely monoclonal gammopathy of undetermined significance (MGUS) and smoldering (asymptomatic) multiple myeloma (SMM) [3, 4]. MM is the second most common hematological malignancy next only to non-Hodgkin's lymphoma, contributing 13% of all malignancies and 1% of all neoplasias [5].

Common front line agents used in the induction therapy of MM are either two drug or three drug combinations of melphalan, dexamethasone, thalidomide, lenalidomide and bortezomib [6]. Bortezomib, a novel dipeptide boronate, was the first therapeutic proteasome inhibitor to be tested in humans. The regimens available for the treatment of relapsed and refractory MM are carfilzomib, bortezomib, thalidomide-dexamethasone and lenalidomide-bendamustine-dexamethasone combination [7]. Pomalidomide, a third generation immunomodulatory agent has also been recently approved by the FDA for the treatment of relapsed and refractory MM [8]. The introduction of novel drugs that target specific intracellular pathways and affect cellular interactions within the tumor microenvironment, have significantly aided in the clinical management of MM patients. However, MM still remains incurable as majority of the patients suffer from relapse after initial response or develop chemoresistance. Moreover, most of the available drugs have severe dose-limiting toxicity including but not limited to bone marrow suppression, peripheral neuropathy, and reactivation of herpes zoster infection [9]. Thus, there remains an unmet need to develop novel therapies for MM treatment.

Resistance to chemotherapy remains a major therapeutic challenge in MM. Several biological mechanisms are implicated in chemoresistance, including multidrug resistance (MDR1/P-glycoprotein [P-gp] or p170), resistance-related proteins (p95 and p110), multidrug resistance-associated protein (p190), proteins implicated in cell detoxification such as glutathione S-transferase and genes affecting DNA structure (topoisomerases) [10]. The precise mechanism underlying chemoresistance in MM is not clear, but one of the main contributors to both chemoresistance and pathogenesis is thought to be activation of master transcription factor NF-κB and dysregulation of apoptosis [11, 12]. Many studies have shown that the NF-κB signaling pathway plays an important role in anti-apoptosis and the drug resistance of tumor cells during chemotherapy. First, many chemotherapeutic drugs and radiotherapy induce NF-κB expression in vitro and in vivo [12, 13]. Second, activation of NF-κB rescues cells from chemotherapy induced apoptosis [14]. Third, induction of chemoresistance and radioresistance is mediated via genes regulated by NF-κB [12]. Fourth, inhibition of NF-κB and NF-κB regulated gene products increases the sensitivity of cancer cells to apoptosis induced by chemotherapeutic agents and to radiation exposure [12, 15]. Activation of NF-κB in MM cells induces proliferation, survival and chemoresistance. When compared to chemosensitive MM cell lines chemoresistant MM cells express higher levels of NF-κB, suggesting a link between NF-κB and development of chemoresistance [15]. Thus targeting deregulated NF-κB activation can be an important strategy pharmacological strategy to overcome chemoresistance in MM patients.

Hence, in the present study we investigated whether thymoquinone (2-isopropyl-5- methyl-1,4-benzoquinone, TQ) can significantly augment the apoptotic effects of bortezomib, both under in vitro system and in vivo conditions using human MM cells and xenograft mouse model. We focused specifically on TQ as it has been shown to possess anti-inflammatory, antioxidant, anti-hypertensive and anti-neoplastic effects in various tumor cells including MM through the modulation of diverse oncogenic molecular targets [12, 16-18]. We report for the first time that TQ inhibited the growth of MM U266 tumors in a subcutaneous mouse model, and that combination of TQ with bortezomib further inhibited tumor growth through the modulation of various markers of proliferation, survival and angiogenesis.

## RESULTS

**Fig 1:**
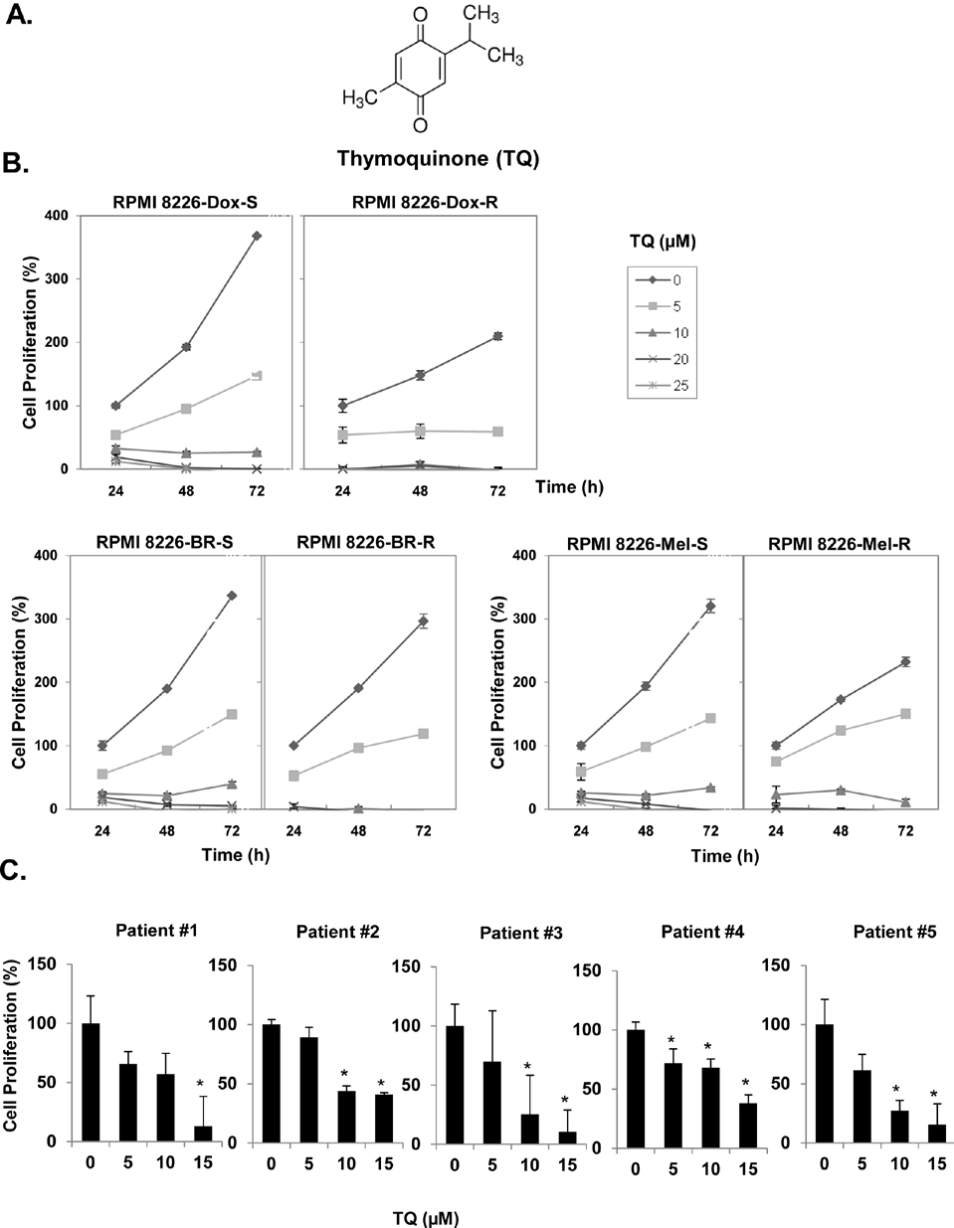
TQ suppresses the proliferation of MM cell lines A, Chemical structure of TQ. B, doxorubicin-sensitive and doxorubicin-resistant RPMI 8266 cells, melphalan-sensitive and melphalan-resistant RPMI 8266 cells, and bortezomib-sensitive and bortezomib-resistant RPMI 8266 cells were plated in triplicate, treated with different concentrations of TQ for 24, 48 and 72 h, and then subjected to MTT assay to analyze proliferation of cells. Each point on line is an average of triplicate value. C, CD138+ cells were isolated from MM patient samples as described in Materials and Methods. The cells were plated in triplicate, treated with different concentrations of TQ and then subjected to MTT assay to analyze proliferation of cells. Columns, mean; bars, SD. *, p < 0.05.

In this study we investigated whether TQ can sensitize MM cells to bortezomib, a clinically used proteasome inhibitor, and the underlying molecular mechanisms involved. To determine the tumor growth inhibition under in vivo conditions, we used a xenograft mouse model. The structure of TQ is shown in Fig. 1A.

### Anti-proliferative effects of TQ on human MM cell lines and patient samples

Anti-myeloma effect of TQ was evaluated by assessing the cell viability of different MM cell lines resistant to various pharmacological drugs. Specifically, we investigated the effect of TQ on RPMI-8226, RPMI-8226-Dox6 (resistant to doxorubicin), RPMI 8226-LR5 (resistant to melphalan), and RPMI-8226-BR (bortezomib resistant) using MTT assay. The data obtained indicated that TQ can significantly suppress the proliferation of all MM cell types examined in a dose- and time-dependent manner (Fig. 1B). We next investigated the effect of TQ on the proliferation of CD138+ cells from 5 patients with MM. The cells were exposed to different concentrations of TQ and then examined for cell proliferation by the MTT method. As shown in Fig. 1C, exposure of CD138+ cells from all 5 MM patients to TQ decreased cell proliferation in a dose-dependent manner.

### TQ potentiates the apoptotic effect of bortezomib in MM cells

**Fig 2:**
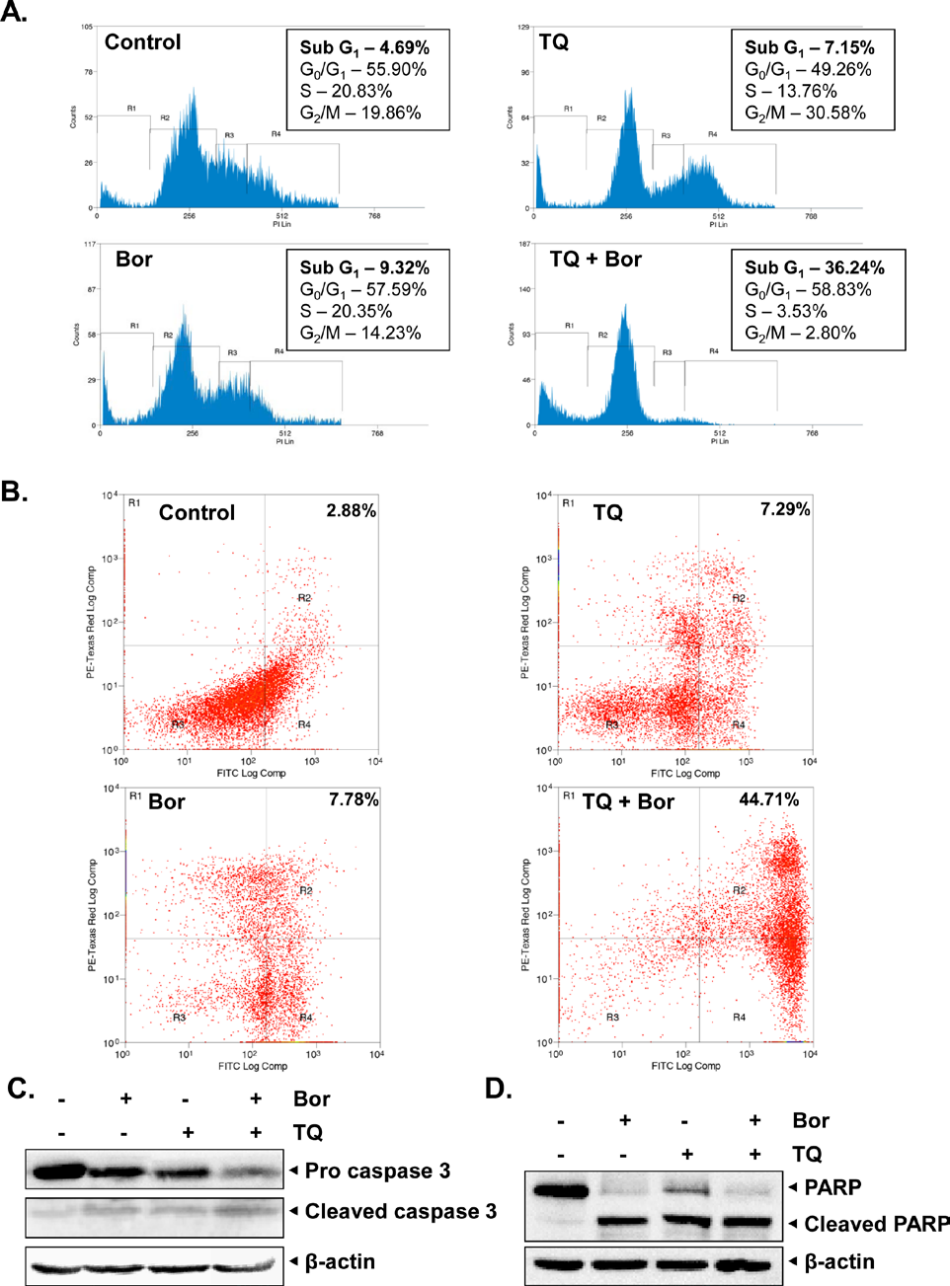
TQ potentiates the apoptotic effect of bortezomib causing accumulation of U266 cells in sub-G1 phase, activates caspase-3 and induce PARP cleavage A, U266 cells were synchronized by serum starvation and then exposed to TQ (5 μM), bortezomib (20 nM) and a combination of both for 24 h at 37°C. The cells were then washed, fixed, stained with PI, and analyzed for DNA content by flow cytometry. B, U266 cells were synchronized by serum starvation and then exposed to TQ (5 μM), bortezomib (20 nM) and a combination of both for 24 h. Cells were incubated with anti-Annexin V antibody conjugated with FITC, followed by PI and then analyzed with a flow cytometry to detect early apoptotic effects. C, U266 cells were treated with TQ (5 μM), bortezomib (20 nM) and a combination of both for 24 h at 37°C. Whole-cell extracts were prepared, separated on SDS-PAGE, and subjected to Western blot analysis using antibody against procaspase-3 and cleaved caspase-3. The same blots were stripped and reprobed with β-actin antibody to show equal protein loading. D, U266 cells were treated with TQ (5 μM), bortezomib (20 nM) and a combination of both for 24 h at 37°C. Whole-cell extracts were prepared, separated on SDS-PAGE, and subjected to Western blot analysis using antibody against PARP. The same blots were stripped and reprobed with β-actin antibody to show equal protein loading. Results typical of two independent experiments are shown.

Bortezomib, an inhibitor of proteasome have been approved for the treatment of patients with MM [15]. We examined whether TQ can potentiate the apoptotic effect of bortezomib in U266 cells. For this, U266 cells were treated with TQ (5 μM), bortezomib (20 nM) and a combination of both, and then examined for apoptosis using cell cycle analysis and annexin V staining by flow cytometry. We found that treatment with a combination of TQ and bortezomib caused statistically significant increase in the accumulation of cell population in the sub-G1 phase as compared to U266 cells treated with TQ or bortezomib alone (Fig. 2A). To further confirm this observation, we used annexin V staining assay, which detects an early stage of apoptosis. The findings of this assay also indicated an enhancement in the apoptotic effects of bortezomib by TQ (Fig. 2B). Cleavage of procaspase-3 to caspase-3 and caspase-3–mediated cleavage of PARP are major characteristic features of apoptosis. In U266 cells treated with a combination of TQ and bortezomib, there was a substantial activation of the effector molecule pro-caspase-3, with a concomitant increase in cleaved form of caspase-3 (Fig. 2C). Moreover, activation of caspase-3 led to the cleavage of a 118 kDa Poly (ADP-ribose) polymerase (PARP) protein into an 85 kDa fragment (Fig. 2D). These results clearly suggested that TQ can augment the apoptotic effect of bortezomib through the activation of caspase-3 in MM cells.

**Fig 3:**
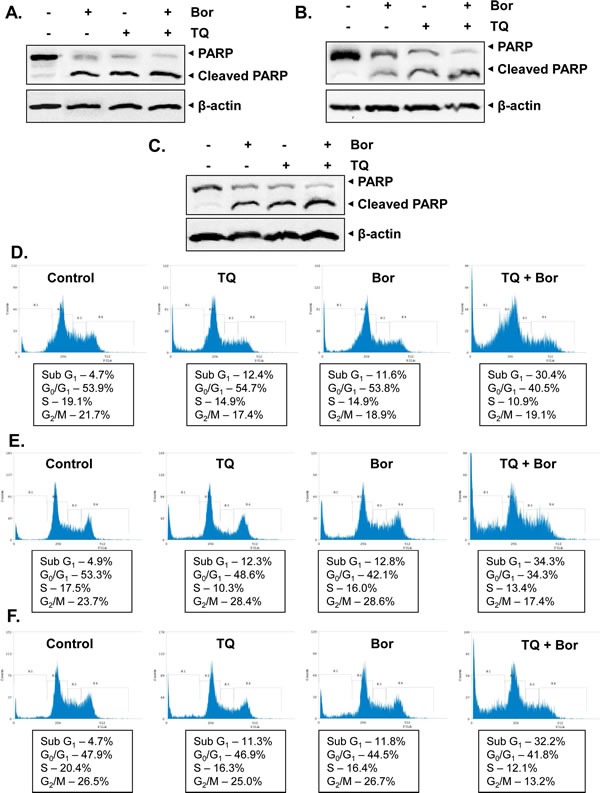
TQ potentiates the apoptotic effect of bortezomib in various MM cell lines A, H929 cells were treated with TQ (5 μM), bortezomib (20 nM) and a combination of both for 24 h at 37°C. Whole-cell extracts were prepared, separated on SDS-PAGE, and subjected to Western blot analysis using antibody against PARP. The same blots were stripped and reprobed with β-actin antibody to show equal protein loading. B, KMS-11 cells were treated with TQ (5 μM), bortezomib (20 nM) and a combination of both for 24 h at 37°C. Whole-cell extracts were prepared, separated on SDS-PAGE, and subjected to Western blot analysis using antibody against PARP. The same blots were stripped and reprobed with β-actin antibody to show equal protein loading. C, RPMI 8226 cells were treated with TQ (5 μM), bortezomib (20 nM) and a combination of both for 24 h at 37°C. Whole-cell extracts were prepared, separated on SDS-PAGE, and subjected to Western blot analysis using antibody against PARP. The same blots were stripped and reprobed with β-actin antibody to show equal protein loading. D, H929 cells were synchronized by serum starvation and then exposed to TQ (5 μM), bortezomib (20 nM) and a combination of both for 24 h at 37°C. The cells were then washed, fixed, stained with PI, and analyzed for DNA content by flow cytometry. E, KMS-11 cells were synchronized by serum starvation and then exposed to TQ (5 μM), bortezomib (20 nM) and a combination of both for 24 h at 37°C. The cells were then washed, fixed, stained with PI, and analyzed for DNA content by flow cytometry. F, RPMI 8226 cells were synchronized by serum starvation and then exposed to TQ (5 μM), bortezomib (20 nM) and a combination of both for 24 h at 37°C. The cells were then washed, fixed, stained with PI, and analyzed for DNA content by flow cytometry. Results typical of two independent experiments are shown.

We also analyzed whether TQ can enhance the apoptotic effect of bortezomib in three other MM cell lines namely, H929, KMS-11 and RPIMI 8226. We found that treatment with TQ in combination with bortezomib substantially increased the PARP cleavage in these three cell lines (Figs 3A-C). We also found that TQ in combination with bortezomib caused significant increase in the cell population in sub-G1 phase as compared to the MM cells treated with TQ or bortezomib alone (Figs. 3D-F). these findings clearly indicate the potential of TQ to augment the apoptotic effects of bortezomib in diverse MM cell lines.

### TQ suppresses MM cell migration and invasion

**Fig 4:**
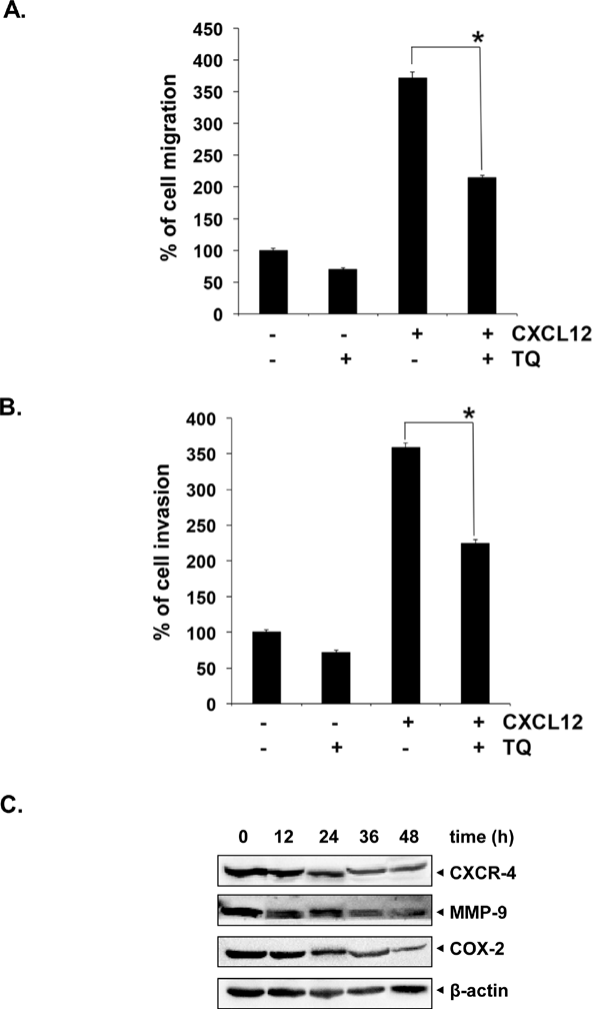
TQ inhibits migration and invasion of MM cell lines A, U266 cells (50x10^4^/well) were plated in 0.3 ml cell culture media with and without 15 μM TQ in the top chambers of 24-well transwell inserts with 8-mm pores. Cell culture medium (600 μl) containing the recombinant human B-cell chemoattractant, CXCL12 (SDF-1α) was added to the bottom chamber and incubated for 12 hours. After incubation, the insert was removed and calcein-AM (5 μM) was added to the wells and fluorescence was measured. Columns, mean; bars, SD. *, p < 0.05. B, U266 cells (50 x10^4^) in suspension were starved in serum-free RPMI-1640 for 3 h, and then loaded onto the Matrigel-coated inserts in the upper chambers of tissue culture inserts placed in 24 well plates. The wells of the plate were filled with 600-μl of 10% FBS-containing cell culture media with 100 ng/ml CXCL12 (SDF-1α). TQ (15 μM) was added with the cells to the upper chamber. Plates were then incubated at 37°C for 12 h. At the end of the incubation period, calcein-AM (5 μM) was added to the wells containing invasive cells, incubated at 37°C for 1 hour and fluorescence was measured. Columns, mean; bars, SD. *, p < 0.05. C, U266 cells were treated with TQ (15 μM) for 0, 12, 24, 36 and 48 h at 37°C. Whole-cell extracts were prepared, separated on SDS-PAGE, and subjected to Western blot analysis using antibody against CXCR4, COX-2 and MMP-9. The same blots were stripped and reprobed with β-actin antibody to show equal protein loading.

The effect of TQ on migration and invasion of U266 cells was also investigated. The addition of TQ to the media with U266 cells in the top chambers of transwell inserts significantly reduced the migration of the cells into the bottom chamber containing CXCL12, a potent lymphocyte chemotactic factor (Fig. 4A). Using an in vitro invasion assay, we also found that CXCL12 significantly induced the invasion of U266 cells across the matrigel coated polycarbonate membrane and that treatment with TQ significantly abrogated the invasive activity (Fig. 4B).

### TQ downregulates the expression of CXCR4, COX-2 and MMP-9 proteins in MM cells

Treatment of U266 cells with TQ was found to downregulate the expression of the chemokine receptor CXCR4, which is specific for CXCL12, the mediator of inflammation, COX-2 and key matrix metalloproteinase MMP-9, which is involved in breakdown of cellular matrix and initiation on invasion. Their expression decreased in a time-dependent manner, with maximum suppression observed at around 48 h (Fig. 4C). The downmodulation in the expression of these proteins may account for observed anti-migratory and anti-invasive potential of TQ.

### TQ potentiates the antitumor effects of bortezomib in a xenograft MM mouse model

**Fig 5:**
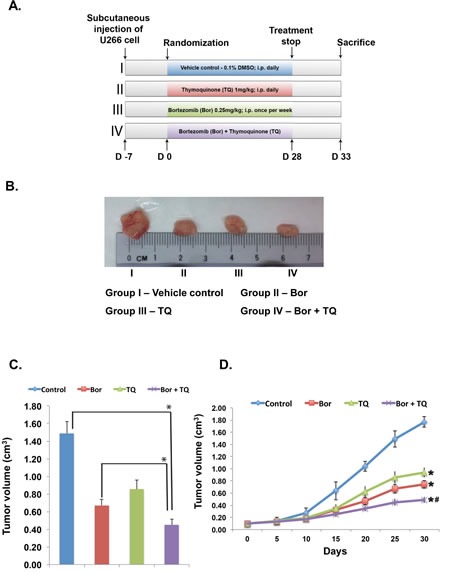
TQ potentiates the anti-tumor activity of bortezomib in xenograft mouse model A, schematic representation of experimental protocol as described in Materials and Methods. Group I was given 0.1% DMSO (100 μL, i.p., daily), group II was given bortezomib (0.25 mg/kg body weight, i.p., once in a week], group III was given TQ (1 mg/kg body weight, i.p., daily), and group IV was given bortezomib (0.25 mg/kg body weight, i.p., once in a week) and TQ (1 mg/kg body weight, i.p., daily) for 4 weeks. B, photographs of tumor tissue dissected from the mice after the end of treatment period. C, tumor volumes in mice measured on the last day of the experiment with Vernier calipers and calculated using the formula V= 4/3πr^3^ (n= 6). Columns, mean; bars, SD. *, p < 0.05. D, tumor volumes measured during the course of experiment and calculated using the formula V= 4/3πr^3^, *, p < 0.05.

We next examined the therapeutic potential of TQ and bortezomib either alone or in combination on the growth of subcutaneously implanted U266 cells in nude mice. The detailed procedures used to establish the U266 model in the athymic nude mice and to treat the mice with TQ, bortezomib, and the combination of TQ and bortezomib is depicted in Fig. 5A. The average tumor volume in bortezomib alone and TQ alone treated groups was significantly smaller than untreated animals (49% and 45%, respectively) as soon as day 15, while at day 30, the average tumor volumes of the bortezomib-alone and the TQ alone treated group were 58% and 47% less than that of the untreated control, respectively. The average tumor volume in bortezomib + TQ -treated group was significantly less (60% at day 15 and 72% at day 30) than in the untreated group (P<0.05) and the bortezomib alone treated group (P<0.05), from day 15 through day 30. Overall, these findings indicated that the combination of bortezomib and TQ was significantly effective in reducing the tumor burden in treated mice (Figs. 5B-D).

### TQ inhibits NF-κB DNA binding activity and the constitutive NF-κB expression in MM tumor tissues

**Fig 6:**
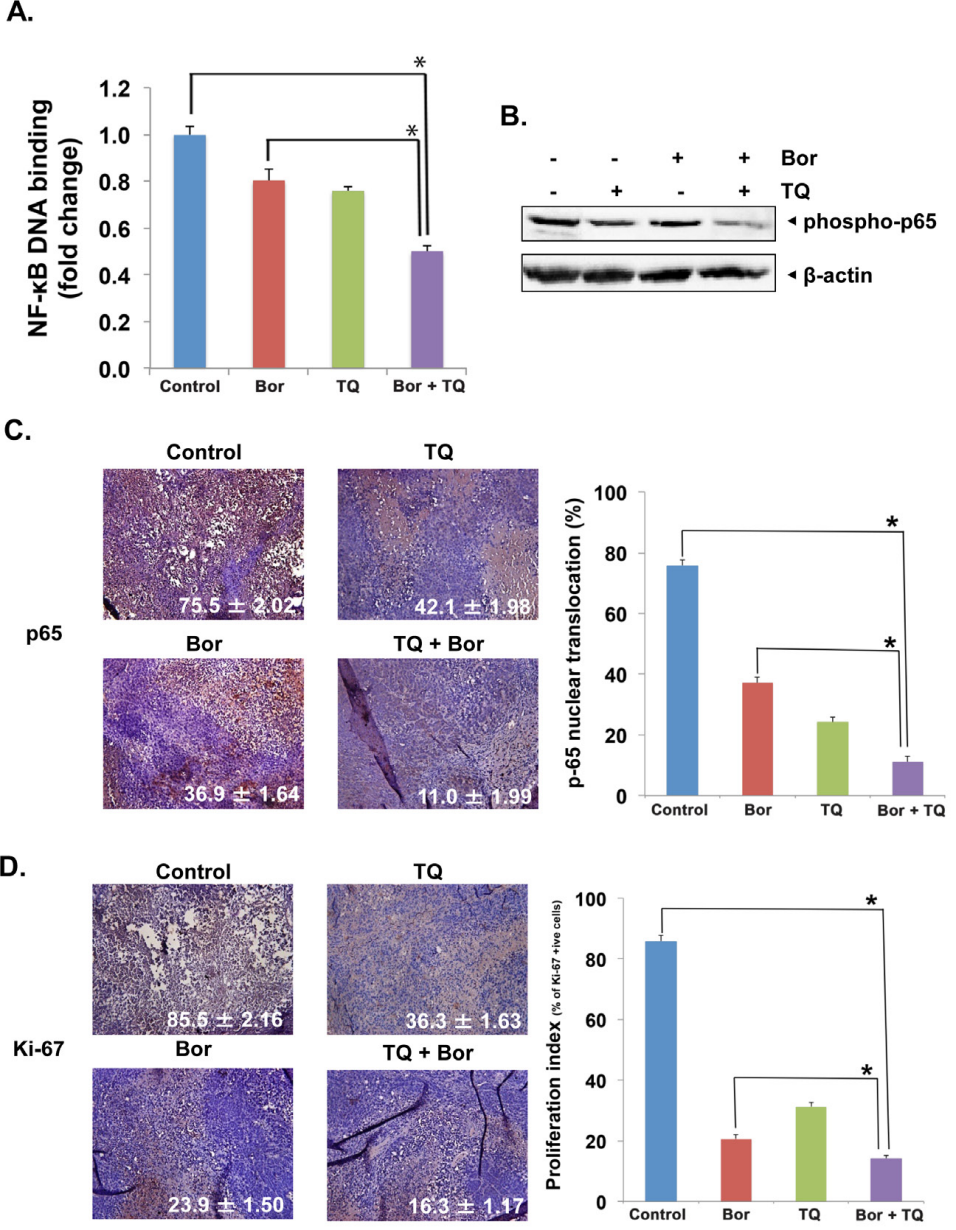
TQ enhances the effect of bortezomib against the expression of NF-κB and inhibit proliferation in MM tumor A, detection of NF-κB activation by DNA-binding assay in tumor tissue samples. Tumor tissues from animals were processed and used for DNA binding assay (Active Motiff) as described in Materials and Methods. The results showed a significant inhibition of NF-κB DNA binding in mice treated with TQ in combination with bortezomib. Columns, mean; bars, SD; *, p < 0.05. B, Western blot analysis using whole-cell extracts from tumor tissue showed the inhibition of NF-κB (p65) by TQ in combination with bortezomib. C, immunohistochemical analysis of NF-κB p65 expression in mice treated with TQ either alone or in combination with bortezomib. Right, quantification of NF-κB p65 positive cells as described in Materials and Methods. Columns, mean; bars, SD; *, p< 0.05. D, left, immunohistochemical analysis of proliferation marker Ki-67 indicates the inhibition of U266 cell proliferation in TQ either alone or in combination with bortezomib treated groups of mice. Right, quantification of Ki-67 positive cells as described in Materials and Methods. Columns, mean; bars, SD; *, p< 0.05.

The pro-inflammatory transcription factor NF-κB plays a pivotal role in the proliferation and survival of various kinds of B-cell tumors, including MM [19, 20]; therefore, the levels of constitutive NF-κB activation in MM tumor tissues was next examined by ELISA-based TransAM NF-κB assay kit. The DNA-binding assay for NF-κB in nuclear extracts from tumor samples showed that TQ in combination with bortezomib significantly suppressed NF-κB activation, while TQ alone and bortezomib alone had much lesser inhibition on NF-κB than the drug combination (Fig. 6A). We also evaluated the effect of TQ and bortezomib on NF-κB (p65) activation by western blot analysis of whole cell extract obtained from MM tissues. Our results clearly showed that the phosphorylation of NF-κB (p65) subunit was substantially reduced in extracts from tumor samples of mice treated with a combination of TQ and bortezomib (Fig. 6B).

### TQ inhibits NF-κB (p65) and Ki-67 expression in MM tumor tissues

NF-κB is known to regulate the expression of numerous genes involved in MM pathogenesis, including growth, survival, immortalization, angiogenesis and metastasis [20, 21], while the nuclear protein Ki-67 is a proliferation index, as it is expressed only by dividing cells. The nuclear localization of p65 and the expression of Ki-67 was examined using an immunohistochemical method described previously [22]. Whether TQ and bortezomib can modulate these markers in MM tumor tissues was examined. We found that both agents significantly reduced the p65 expression as compared with control group individually and the combination was most effective (p < 0.05 when compared with TQ alone; Fig. 6C). Fig. 6D shows that both TQ (p < 0.05) and bortezomib (p < 0.05) alone significantly downregulated the expression of Ki-67 in MM tumor tissues and the combination of the two was even more effective (p < 0.05).

### TQ inhibits expression of VEGF and Bcl-2 in tumor tissues

**Fig 7:**
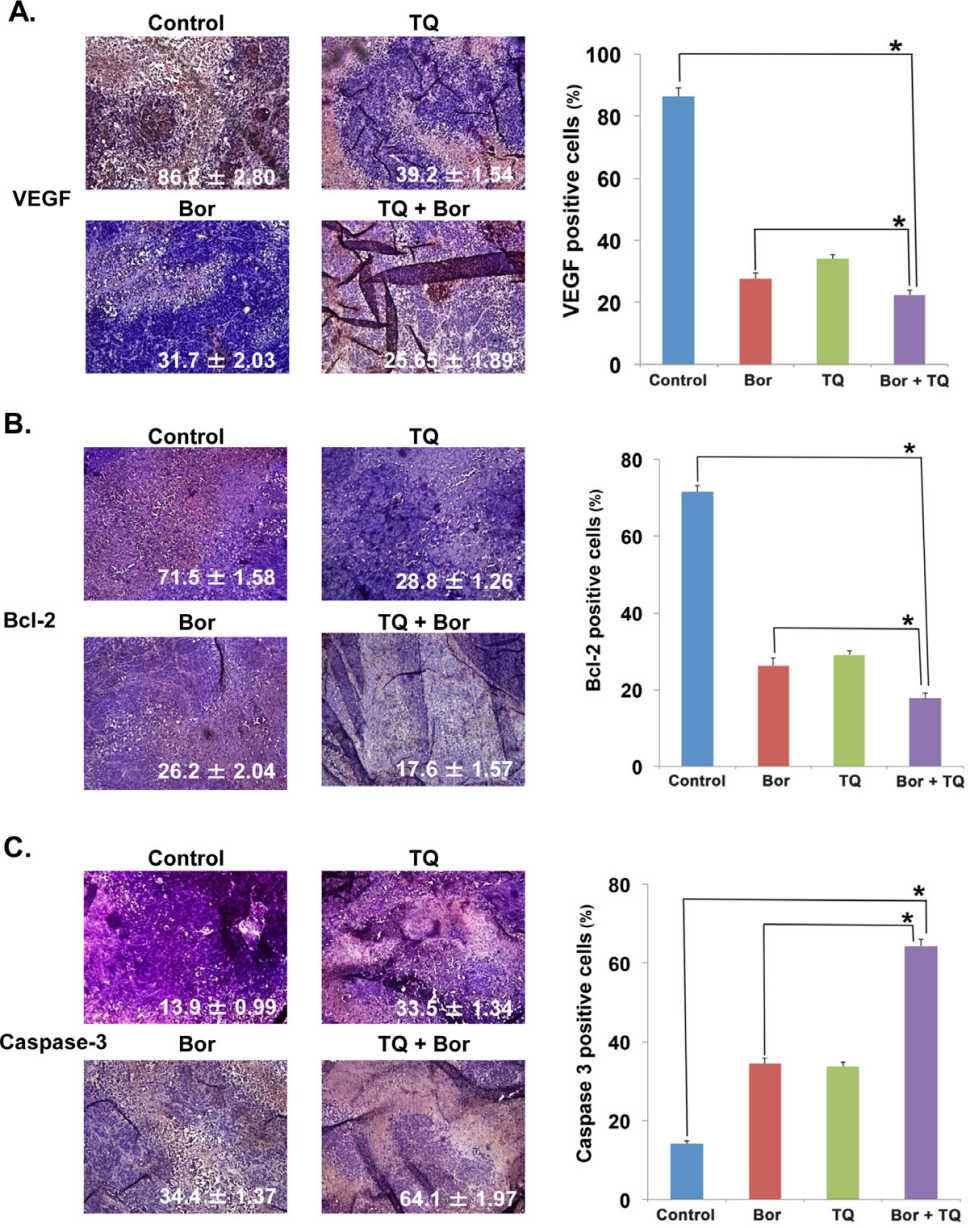
TQ enhances the effect of bortezomib against MM tumor cell proliferation A, immunohistochemical analysis of the pro-angiogenic growth factor VEGF expression in mice treated with TQ either alone or in combination with bortezomib. Right, quantification of VEGF positive cells as described in Materials and Methods. Columns, mean; bars, SD; *, p < 0.05. B, immunohistochemical analysis of the survival protein Bcl-2 expression in mice treated with TQ either alone or in combination with bortezomib. Right, quantification of Bcl-2 positive cells as described in Materials and Methods. Columns, mean; bars, SD; *, p < 0.05. C, immunohistochemical analysis of caspase-3 expression in mice treated with TQ either alone or in combination with bortezomib. Right, quantification of caspase-3 positive cells as described in Materials and Methods. Columns, mean; bars, SD; *, p < 0.05

VEGF, another protein regulated by NF-κB plays an important role in angiogenesis and growth of MM cells, so we next examined its expression in MM tumor samples. Our results showed that TQ alone and bortezomib alone significantly decreased the expression of VEGF in tumor tissue (Fig. 7A). However, TQ + bortezomib combination was more effective in reducing VEGF expression than either agent used alone (p < 0.05 versus TQ alone). The Bcl-2 protein, regulated by NF-κB is also highly expressed in myeloma patients and in vitro studies have shown its role in the regulation of chemosensitivity [23, 24]. As shown in Fig. 7B, the expression of Bcl-2 in MM tumor tissue was significantly downregulated in mice treated with either TQ or bortezomib alone. However, the downregulation was more substantial in mice treated with a combination of TQ and bortezomib. Next, to determine whether TQ decreases MM tumor growth by inducing apoptosis, we examined the caspase-3-positive cells in tumors obtained from mice. Our results showed that TQ alone and bortezomib alone caused a similar level of increase in caspase-3 expression. Interestingly, we found that the TQ + bortezomib combination was substantially more effective in inducing caspase-3 activation rather than either agent used alone (Fig. 7C; p< 0.05 versus vehicle and TQ alone).

### TQ reduces serum levels of IL-6 and TNF- α in MM tumor bearing mice

**Fig 8:**
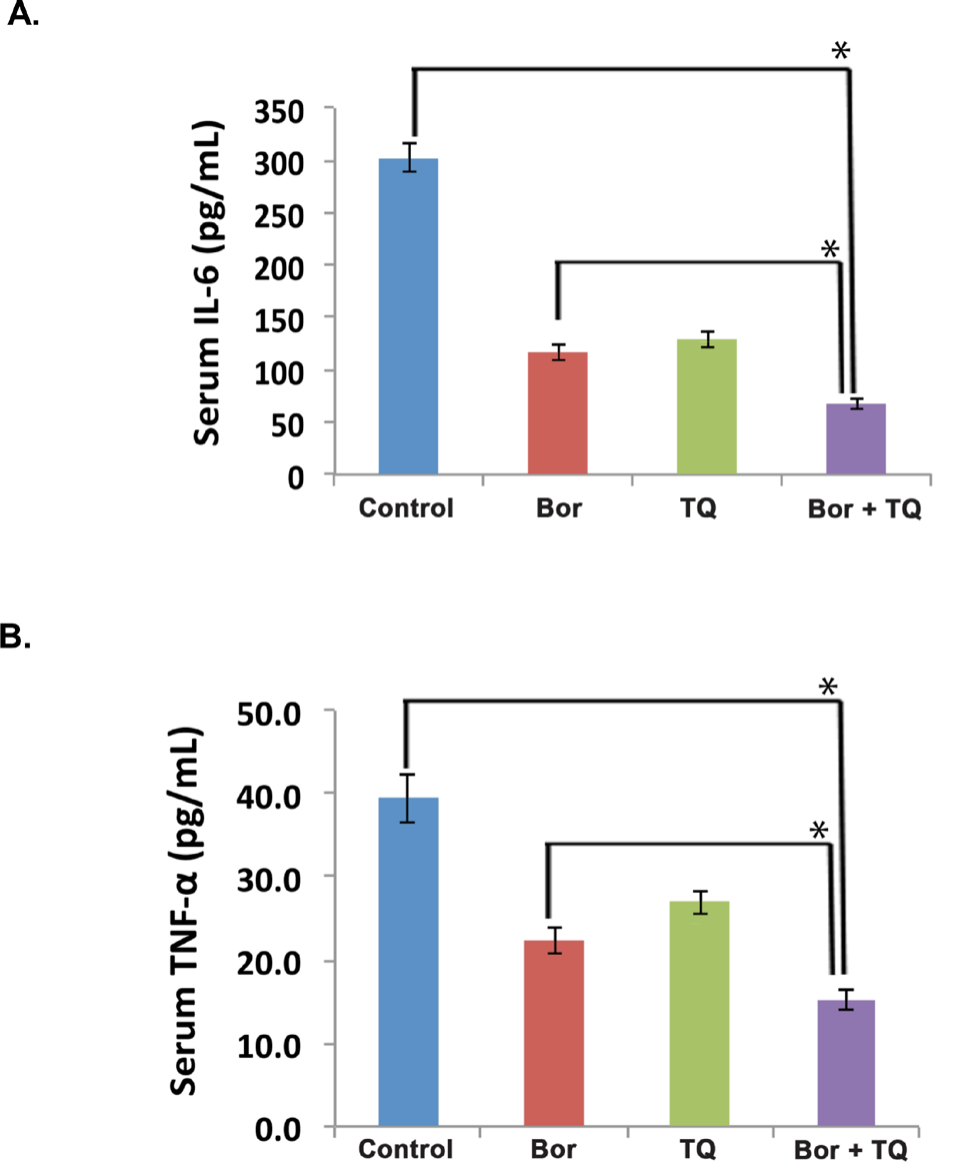
TQ modulates serum levels of IL-6 and TNF-α in MM tumor bearing mice A, All four groups of mice were treated as described in Materials and Methods. Sandwich ELISA assay was performed as per manufacturers' instruction R&D systems (Minneapolis, USA) to determine the levels of IL-6. *Statistical significance (p < 0.05). B, All four groups of mice treated as described in Materials and Methods. Sandwich ELISA assay was performed as per manufacturers' instruction R&D systems (Minneapolis, USA) to determine the levels of TNF-α. *Statistical significance (p < 0.05).

The pro-inflammatory cytokines IL-6 and TNF-α, are considered as major biomarkers of inflammation. Hence, we also analyzed the effect of TQ and bortezomib on the levels of these two cytokines in serum samples obtained from mice. MM tumor-bearing mice treated with TQ and bortezomib alone or in combination were sacrificed at the end of each treatment protocol. Blood was collected via cardiac puncture and serum separated and used to evaluate the levels of IL-6 and TNF-α using an ELISA kit. Interestingly, levels of both IL-6 and TNF-α level was found to be substantially higher in untreated tumor bearing mice. TQ and bortezomib alone treatment significantly decreased serum IL-6 when compared to control, while the TQ + bortezomib treated mice had 77% reduction in serum IL-6 compared to untreated control (p < 0.05) (Fig.8A). Similarly serum levels of TNF-α was significantly reduced in mice treated with a combination of TQ and bortezomib (61%, p < 0.05 versus untreated control) (Fig.8B).

## DISCUSSION

The aim of this study was to determine whether TQ could augment the apoptotic effect of bortezomib in MM cell lines and xenograft mouse model and if so through what mechanisms. We found that TQ inhibited the proliferation of various MM cell lines regardless of their sensitivity/resistance to the conventional chemotherapeutic agents such as doxorubicin, melphalan and/or targeted therapies such as bortezomib. TQ also inhibited the survival of CD138+ plasma cells obtained from MM patients in a dose dependent manner. It potentiated the apoptotic effects of bortezomib in MM cells as evidenced by increased accumulation of cells in the sub-G1 phase leading to the caspase-3 activation and PARP cleavage. Our results also show that enhancement of apoptotic effects of bortezomib induced by TQ is not limited to one MM cell line, but can be observed in several MM cell lines. We further observed that in a xenograft mouse model TQ alone and in combination with bortezomib effectively suppressed the growth of subcutaneous tumor of MM cells through the suppression of NF-κB activation. This correlated with down-regulation of various proliferative, anti-apoptotic, angiogenic and inflammatory biomarkers regulated by NF-κB in MM tumor tissues.

The pathogenesis of MM involves characteristic genetic abnormalities and the interaction between the clonal plasma cells and the bone marrow microenvironment [25]. Unlike other hematological malignancies and similar to solid tumors, the genetic abnormalities of MM are heterogeneous and the transformation is not driven by a single gene mutation. Thus developed MM, through complex genetic and epigenetic events further progresses and is no longer dependent on bone marrow microenvironment for survival leading to extramedullary MM. Adhesion of MM cells to the stromal cells induces the latter to secrete IL-6. IL-6 is the main growth factor for the MM cells. In turn IL-6 then induces janus kinase (JAK) / signal transducer and activator of transcription 3 (STAT3), PI3K/Akt and mitogen-activated protein kinase survival pathways in MM cells [20, 26]. We had previously reported that TQ can indeed inhibit proliferation and induce apoptosis in MM cells through the suppression of signal transducer and activator of transcription 3 (STAT3) activation pathway [12]. However, the effect of TQ on drug resistant MM cell lines, patient samples and xenograft mouse model was not investigated in that report. Moreover, we have also reported NF-κB inhibitory potential of TQ in diverse tumor cell lines previously [17], which may account for its observed anti-proliferative/apoptotic effects as observed in the present study.

CXCL12 is a member of CXC family of cytokines and its cognate receptor is CXCR4. It has been implicated in progression, migration, invasion and metastasis of various cancers [27]. BMSCs secrete this chemokine, with the MM cells from the patient sample and MM derived cell lines expressing the cognate receptors [28]. This chemokine mediates the secretion of IL-6 and VEGF and induces proliferation, migration and inhibits dexamethasone induced cell death [29]. Accordingly, CXCL12 induces invasion and CXCR4 antagonists can negatively regulate CXCL12-induced invasion. We noted that TQ can significantly inhibit CXCL12 induced chemotactic movement as well as invasive potential in MM cell lines which may be mediated by the downregulation of CXCR4 expression as observed by western blot analysis.

MMPs belong to a family of proteases, capable of degrading all kinds of extracellular matrix proteins [30]. Culture supernatants of bone marrow derived stromal cells from MM patients have been found to have higher levels of MMP than control samples [31]. Moreover, endothelial cells can secrete hepatocyte growth factor, which then acts on MM cells to stimulate the secretion of MMP-9 [32]. Adequate inhibition of various MMPs by a broad spectrum MMP inhibitor SC-964 can suppress angiogenesis, reduces tumor load and osteolytic lesions [33]. Our study showed that TQ could substantially inhibit the expression of MMP-9 in MM cell lines, which may account for its observed anti-migratory/anti-invasive effects in MM cells.

In the present study, we also investigated for the first time the potential effect of TQ alone or in combination with bortezomib on MM growth in a xenograft mouse model. We found that the intraperitoneal administration of TQ alone could inhibit the growth of MM tumors in the xenograft model. Bortezomib also induced a significant inhibition of tumor growth. But when the two agents were used in combination, they were found to be much more effective in significantly suppressing the growth of MM tumor. When examined for the mechanisms, we observed that the activation of transcription factor NF-κB was substantially attenuated by these two drugs when used in combination. NF-κB has been casually implicated in progression of various types of tumors [14]. MM patient samples show a constitutive activation of NF-κB to variable degree. 15-20% of MM samples and 40% of the cell lines show mutations that lead to constitutive activation of NF-κB pathway [34]. Activation of NF-κB in MM cells induces proliferation, survival and chemoresistance. When compared to chemosensitive MM cell lines chemoresistant MM cells express higher levels of NF-κB, suggesting a link between NF-κB and development of chemoresistance [12, 35]. Moreover, dexamethasone induced apoptosis is associated with a decrease in the NF-κB DNA binding activity [35]. In agreement with these previous reports, we also found that MM tumor samples had elevated levels of basal NF-κB expression as well as increased activation as observed by DNA binding and western blot analysis. Previous studies has shown that NF-κB activation was inhibited when bortezomib was added, suggesting that bortezomib can sensitize MM cells to alkylating agents by inhibiting NF-κB activation [36]. In our study we found that bortezomib can decrease the NF-κB activation in MM tumor samples, but the TQ combination had a significant inhibition in NF-κB activation even when compared to bortezomib alone treatment. This may explain the chemosensitizing effects of TQ as observed here and also reported previously [12, 17].

VEGF is an important signaling protein regulated by NF-κB that stimulates the formation of new blood vessels, through vasculogenesis and angiogenesis [37]. Dysregulation of VEGF has been shown to be a major contributor to tumor angiogenesis as well, promoting tumor growth, invasion and metastasis [38]. Significantly elevated levels of VEGF are observed in a variety of hematological malignancies [39, 40]. VEGF protein was found in malignant cells from 75% of MM patients studied [41] and increased serum levels of VEGF have been correlated with a poor prognosis in patients with advanced stages of MM [20, 42]. Thus the observed significant downregulation of VEGF expressed in the tumor tissues from mice treated with a combination of TQ and bortezomib may contribute to the anti-tumor activity through the inhibition of angiogenic pathways that are essential for the tumor growth.

Overexpression of anti-apoptotic molecules of Bcl-2 family has been linked to chemoresistance in MM [20, 24]. Prior studies has shown that the activation of NF-κB can enforce the ectopic expression of Bcl-2 in MM cells conferring resistance to dexamethasone induced apoptosis through activation of survival pathways [43]. However TQ in combination with bortezomib was found to suppress the expression of Bcl-2 in MM tissues, which may also account for its tumor inhibitory effects. This decline in the survival signaling pathway contributed to the increased apoptosis in the tumor tissues as evidenced by increase in caspase-3 expression by immunohisrochemistry. IL-6, a pleiotropic cytokine, is one of the major growth factors in MM [44]. IL-6 induces survival of MM cell through activation of STAT3, which upregulates anti-apoptotic proteins Bcl-xL and Mcl-1 and cell cycle proteins like cyclin D1, and cyclin E [20, 24]. The fact that TQ can abrogate IL-6 induced STAT3 activation has already been reported by our group previously [12]. In the present study, we noted that MM bearing mice treated with TQ in combination with bortezomib produced a significant inhibition in the production of IL-6, when compared to both vehicle control and bortezomib alone treated groups, indicating that the combination is quite effective in blocking the IL-6 mediated growth and survival of MM. TNF-α is another important growth factor secreted from MM cells to act on BMSCs to stimulate the secretion of IL-6. TNF-α induces the expression of adhesion molecules on both MM cells and BMSCs [45]. TNF-α have been reported to be involved in the control of VEGF production by MM cells [46]. TNF-α also participates in transendothelial migration of MM cells by acting via TNF-R2 and upregulating the secretion of MCP-1 (monocyte chemoattractant protein-1) in MM cells [47]. Interestingly, TNF-α blockers such as thalidomide and other immunomodulatory agents have exhibited significant anti-myeloma activity [48]. We observed that TQ in combination with bortezomib produced significant reduction in the serum levels of TNF-α, which can contribute to the anti-tumor activity. Also since both these cytokines are regulated by NF-κB, inhibitory effects of TQ on NF-κB may explain its potential suppressive effects on the serum levels of IL-6 and TNF-α.

TQ has been extensively studied so far in diverse tumor models without exhibiting any significant toxic effects to normal cells [16]. However, TQ has never been tested in humans before either alone or in combination with targeted therapies and hence its clinically relevant doses are not clear as yet. Overall, the ability of TQ to inhibit cell proliferation, migration, invasion and potentiate the apoptotic effects of bortezomib while suppressing NF-κB activation and its regulated gene products, provides a sound rationale to test this agent to enhance treatment efficacy, reduce toxicity, and overcome chemoresistance of relapsed or refractory MM.

## MATERIALS AND METHODS

### Reagents

Thymoquinone, MTT, Tris, glycine, NaCl, SDS, BSA and β-actin antibody were obtained from Sigma-Aldrich (Missouri, USA). TQ was dissolved in dimethyl sulfoxide (DMSO) as a 50 mM stock solution and stored at −20°C for the experiments. Further dilution was done in cell culture medium, so that the final DMSO concentration was less than 0.1%. RPMI 1640, 0.4% trypan blue vital stain and antibiotic-antimycotic mixture were obtained from Invitrogen (California, USA). Fetal bovine serum (FBS) was purchased from BioWest (Miami, FL, USA). Antibodies to p65, COX-2, Bcl-2, Ki-67, VEGF, pro-caspase-3, cleaved caspase-3, PARP, MMP-9, CXCR4, goat anti-rabbit-horse radish peroxidase (HRP) conjugate and goat anti-mouse HRP were obtained from Santa Cruz Biotechnology (CA, USA). ELISA kits for mouse IL-6 and TNF-α were purchased from R&D Systems Inc (Minneapolis, USA). Nuclear extraction and DNA binding kits was obtained from Active Motif (Carlsbad, CA).

### Cell lines

Human MM cell lines U266, H929, KMS, RPMI-8226, RPMI-8226-Dox-6 (doxorubicin-resistant clone), RPMI-8226-LR-5 (a melphalan-resistant clone) were kindly provided by Dr Leif Bergsagel from Mayo Clinic, Arizona, USA. RPMI-8226-BR (bortezomib resistant clones) was kindly provided by Dr Jacqueline Cloos from Vrije Universiteit Medical Center, Amsterdam, Netherlands. H929 cells were cultured in RPMI 1640 medium containing 2-mercaptoethanol at a final concentration of 0.05 mM, supplemented with 10% fetal bovine serum (FBS). All the other human MM cells were cultured in RPMI 1640 supplemented with 10% fetal bovine serum (FBS) containing 1 X antibiotic-antimycotic solution.

### Cell proliferation assay

The anti-proliferative effect of TQ against various MM cell lines and patient samples was determined by the MTT dye uptake method. Primary MM patient cells were obtained from bone marrow aspirates of patients after informed consent and with ethical approval from the NUS IRB. Peripheral blood mononuclear cells were separated with RBC lysis buffer and subsequently CD138+ plasma cells were isolated using magnetic cell sorting with CD138 easysep magnetic nanoparticles (Stemcells Technologies, Singapore) according to manufacturer's instructions. Purified CD138+ patient cells were grown in IMDM, Glutamax (Gibco, Invitrogen), supplemented with 20% fetal bovine serum (FBS), 100 U/mL penicillin and 100 µg/mL streptomycin, 10ng/mL of IL-6 (Militenyi Biotech,Surrey UK) and 100ng/mL of rhIGF-1(R&D Systems, Oxford, UK). All cells were grown at 37°C in a humidified atmosphere with 5% CO_2_. Briefly, the cells (5 × 10^3^/well) were incubated in triplicate in a 96-well plate in the presence or absence of indicated concentrations of TQ in a final volume of 0.2 ml for different time intervals at 37 °C. Thereafter, 20 μl of MTT solution (5 mg/ml in PBS) was added to each well. After a 4-h incubation in the dark at 37 °C, 0.1 ml of lysis buffer (20% SDS, 50% dimethylformamide) was added and incubated for 2 h at 37 °C, followed by measurement of optical density at 570 nm by Tecan plate reader (Durham, NC).

### Flow cytometric analysis

To determine whether TQ can potentiate the apoptotic effect of bortezomib, MM cells were first synchronized by serum starvation and then exposed to TQ (5 μM), bortezomib (20 nM) and a combination of both for 24 h. Thereafter cells were washed, fixed with 70% ethanol, and incubated for 30 min at 37°C with 0.1% RNase A in PBS. Cells were washed again, resuspended, and stained with PBS containing 25 μg/mL propidium iodide (PI) for 30 min at room temperature. Cell distribution across the cell cycle was analyzed with a CyAn ADP flow cytometer (Dako Cytomation).

### Annexin V assay

The procedure of annexin V-PI staining was carried out according to the manufacturer's protocol (Invitrogen). U266 cells (5× 10^5^/mL) were treated with TQ (5 μM), bortezomib (20 nM) and a combination of both for 24 h. After the incubation, the cells were washed with PBS, and resuspended in FITC-conjugated annexin V and PI-containing binding buffer for 15 min in dark. Samples were then analyzed immediately by flow cytometer.

### Western blot analysis

For detection of proteins, TQ-treated cells were harvested and lysed in whole cell lysis buffer (20 mM Tris (pH 7.4), 250 mM NaCl, 2 mM EDTA (pH 8.0), 0.1% Triton X-100, 0.01 mg/ml aprotinin, 0.005 mg/ml leupeptin, 0.4 mM PMSF, and 4 mM NaVO4). Lysates were then spun at 14,000 rpm for 10 min to remove insoluble material and stored at −80°C for later use. The protein content in the lysates was measured by Bio-Rad Protein Assay Dye Reagent Concentrate (Bio-Rad, CA) and equal quantity of protein was resolved on a 10% SDS gel. After electrophoresis, the proteins were electrotransferred to a nitrocellulose membrane, blocked with 5% nonfat milk, and probed with appropriate antibody overnight at 4°C. The blot was washed, exposed to HRP-conjugated secondary antibodies for 1 h, and finally examined by chemiluminescence (ECL; GE Healthcare, Buckinghamshire, UK).

### Migration assay

U266 cells (50x10^4^/well) were plated in 0.3 ml cell culture media with and without 15 μM TQ in the top chambers of 24-well transwell inserts with 8-mm pores. Cell culture medium (600 μl) containing the Recombinant Human B-cell chemoattractant, CXCL12 (SDF-1α) (100 ng/ml; Prospec, Ness-Ziona, Israel) was added to the bottom chamber and incubated for 12 h. After incubation, the insert was removed and calcein-AM (5 μM) was added to the wells. The cells were incubated at 37°C for 1 hour to allow the cells to internalize calcein-AM. The fluorescence was measured (485 nm excitation, 520 nm emission). A standard curve was generated with cells ranging from 50000-1000 cells using calcein-AM under same condition and cells number calculated from relative fluorescence of treated samples.

### Invasion assay

An invasion assay was performed with U266 cells in 24-well plates with polycarbonate membranes (ThinCert 8-μm pore size tissue culture insert, Greiner Bio-one, NC, USA). Briefly the upper chambers were coated with 50-μl Matrigel (Becton Dickinson, Bedford, MA, USA) in advance according to the directions of the manufacturer. U266 cells (50 x10^4^) in suspension were starved in serum-free RPMI-1640 for 3 h, and then loaded onto the Matrigel-coated inserts in the upper chambers. The wells of the plate were filled with 600-μl of 10% FBS-containing cell culture media with 100 ng/ml CXCL12 (SDF-1α) (Prospec, Ness-Ziona, Israel). TQ (15μM) was added with the cells to the upper chamber. Plates were then incubated at 37°C for 12 h. At the end of the incubation period, calcein-AM (5μM) was added to the wells containing invasive cells. The cells were incubated at 37°C for 1 hour to allow the cells to internalize calcein-AM. The fluorescence was measured (485 nm excitation, 520 nm emission). A standard curve was generated with cells ranging from 50000-1000 cells using calcein-AM under same condition and cells number calculated from relative fluorescence of treated samples.

### Xenograft tumor model

All the procedures involving animals were reviewed and approved by National University of Singapore Institutional Animal Care and Use Committee. Five-week-old athymic nu/nu male Balb/c mice (Biological Resource Centre, Biopolis, Singapore) were implanted subcutaneously in the right flank with U266 cells (2 × 10^6^ cells/100 μL of PBS/Matrigel). When tumors have reached 0.25 cm in diameter, the mice were randomized into the following treatment groups (n = 6/group): (i) untreated control (DMSO (0.1%; v/v), 100 μL intraperitoneally); (ii) bortezomib (0.25 mg/kg of body weight, suspended in DMSO (0.1%; v/v), intraperitoneally, weekly once; (iii) TQ (1 mg/kg of body weight, suspended in DMSO (0.1%; v/v), intraperitoneally, daily); and (iv) TQ (1 mg/kg of body weight, daily) + bortezomib (0.25 mg/kg of body weight weekly once), intraperitoneally. The treatment was continued for 4 weeks, and then the mice were maintained without any drug for 5 more days. The body weights and tumor sizes were recorded every five days, and the tumor size was determined by a Vernier caliper measurement. The animals were euthanized at the end of the therapy and blood collected by heart puncture. Primary tumors were excised and the final tumor volume was measured as V= 4/3πr^3^, where r is the mean radius of the 3 dimensions (length, width, and depth). Half of the tumor tissue was fixed in formalin and embedded in paraffin for immunohistochemistry and routine hematoxylin and eosin (H&E) staining. A small portion of tumor (75-100 mg/mouse) was used for nuclear extraction. The remaining tumor tissue was snap frozen in liquid nitrogen and stored at−80°C.

### Preparation of nuclear extract from tumor samples

Nuclear extract was prepared from the MM tumor tissues using nuclear extraction kit from Active Motiff (Carlsbad, CA), according to manufacturer's protocol. Tumor tissue (75-100 mg/mouse) from control and treated mice were minced in 0.3 ml ice-cold 1X hypotonic buffer supplemented with DTT and detergent and homogenized using a Dounce homogenizer, and then centrifuged at 850 × g at 4°C for 10 min. The supernatant was transferred into a pre-chilled microcentrifuge tube and stored for later use. The cell pellet was resuspend cells in 500 μL 1X hypotonic buffer and incubate for 15 min on ice. Detergent (25 μL) was added, vortexed and the centrifuged for 30 seconds at 14,000 x g in a microcentrifuge pre-cooled at 4°C. The resulting nuclear pellet was suspended in 50 μL complete lysis buffer and incubated on ice for 30 min with intermittent mixing. The suspension was then centrifuged at 14,000 × g at 4°C for 30 min. The supernatant (nuclear extract) was collected and stored at −70°C until use.

### NF-κB DNA-binding assay

To determine NF-κB activation in MM tumor samples, we conducted DNA-binding assay using TransAM NF-κB Kit (Active Motiff), according to the manufacturer's instructions. Briefly, 20 μg of nuclear proteins were added into a 96-well plate coated with an unlabeled oligonucleotide containing the consensus binding site for NF-κB (5′-GGGACTTTCC-3′) and incubated for 1 hour. The wells were washed and incubated with antibodies against NF-κB p65 subunit for 1 hour. An HRP-conjugated secondary antibody was then applied to detect the bound primary antibody and it provided the basis for colorimetric quantification. The enzymatic product was measured at 450 nm with a reference wavelength of 655 nm by microplate reader (Tecan Systems).

### Immunohistochemical analysis of tumor tissues

Solid tumors from control and the various treated groups were fixed with 10% phosphate-buffered formalin, processed, and embedded in paraffin. The sections were cut and deparafinized in xylene, dehydrated in graded alcohol, and finally hydrated in water. Antigen retrieval was conducted by boiling the slide in 10 mmol/L sodium citrate (pH 6.0) for 30 min. Immunohistochemistry was conducted following the manufacturer's instructions (Dako LSAB Kit). Briefly, endogenous peroxidases were quenched with 3% hydrogen peroxide. Nonspecific binding was blocked by incubation in the blocking reagent in the LSAB Kit (Dako) according to the manufacturer's instructions. Sections were incubated overnight with primary antibodies as follows: anti-p65, anti-Ki-67, anti-VEGF, anti-Bcl-2 and anti-caspase3 (each at 1:100 dilutions). The slides were subsequently washed several times in TBS with 0.1% Tween-20 and were incubated with biotinylated linker for 30 minutes, followed by incubation with streptavidin conjugate provided in the LSAB Kit according to the manufacturer's instructions. Immunoreactive species were detected using 3,3′-diaminobenzidine tetrahydrochloride as a substrate. The sections were counterstained with Gill's hematoxylin and mounted under glass cover slips. Images were taken using an Olympus BX51 microscope (magnification, ×20). Positive cells (brown) were quantitated using the Image-Pro plus 6.0 software package (Media Cybernetics, Inc.). A total of five fields were examined and counted from three tumors of each treatment group. The values were initially subjected to one-way ANOVA and then later compared among groups using an unpaired Student's t test, with p < 0.05 considered to be significant.

### Analysis of serum levels of IL-6 and TNF-α

The serum levels of IL-6 and TNF-α were determined using ELISA kits R&D systems (Minneapolis, USA) as per manufacturer's instructions. Blood collected from mice was incubated for 30 minutes at room temperature and then centrifuged at 1500 ×g at 4°C for 10 min to collect the serum. Briefly 50 μL assay diluent was added to the wells of antibody coated microplates followed by 50 μL sample/Standard. The plates were allowed to incubate at room temperature for 2 hours. The contents of the well was aspirated and then washed with 1X wash buffer for 5 times. Mouse IL-6/ TNF-α antibody (100 μL) was added to all wells and allowed to incubate for 2 h at room temperature. The aspiration/washing was repeated and 100 μL of substrate solution added to each well, followed by incubation at room temperature for 30 min. Stop solution (100 μL) was added to each well, mixed and then optical density measured using a microplate reader set to 450 nm.

### Statistical analysis

Data are expressed as the mean ± S.D. In all figures, vertical error bars denote the S.D. The significance of differences between groups was evaluated by Student's t-test and one way analysis of variance, (ANOVA) test. A p value of less than 0.05 was considered statistically significant.

## References

[1] Mirandola L, Yu Y, Chui K, Jenkins MR, Cobos E, John CM, Chiriva-Internati M (2011). Galectin-3C inhibits tumor growth and increases the anticancer activity of bortezomib in a murine model of human multiple myeloma. PloS one.

[2] Kuehl WM, Bergsagel PL (2012). Molecular pathogenesis of multiple myeloma and its premalignant precursor. The Journal of clinical investigation.

[3] Landgren O, Kyle RA, Pfeiffer RM, Katzmann JA, Caporaso NE, Hayes RB, Dispenzieri A, Kumar S, Clark RJ, Baris D (2009). Monoclonal gammopathy of undetermined significance (MGUS) consistently precedes multiple myeloma: a prospective study. Blood.

[4] Kyle RA, Remstein ED, Therneau TM, Dispenzieri A, Kurtin PJ, Hodnefield JM, Larson DR, Plevak MF, Jelinek DF, Fonseca R (2007). Clinical course and prognosis of smoldering (asymptomatic) multiple myeloma. New England Journal of Medicine.

[5] Siegel R, Naishadham D, Jemal A (2013). Cancer statistics, 2013. CA: A Cancer Journal for Clinicians.

[6] Rajkumar SV (2011). Treatment of multiple myeloma. Nat Rev Clin Oncol.

[7] Kortuem KM, Stewart AK (2013). Carfilzomib. Blood.

[8] Elkinson S, McCormack PL (2013). Pomalidomide: First Global Approval. Drugs.

[9] Podar K, Tai Y-T, Hideshima T, Vallet S, Richardson PG, Anderson KC (2009). Emerging therapies for multiple myeloma.

[10] Rossi J (1995). Chemoresistance and multiple myeloma: from biological to clinical aspects. Stem cells (Dayton, Ohio).

[11] Bhardwaj A, Sethi G, Vadhan-Raj S, Bueso-Ramos C, Takada Y, Gaur U, Nair AS, Shishodia S, Aggarwal BB (2007). Resveratrol inhibits proliferation, induces apoptosis, and overcomes chemoresistance through down-regulation of STAT3 and nuclear factor-κB–regulated antiapoptotic and cell survival gene products in human multiple myeloma cells. Blood.

[12] Li F, Rajendran P, Sethi G (2010). Thymoquinone inhibits proliferation, induces apoptosis and chemosensitizes human multiple myeloma cells through suppression of signal transducer and activator of transcription 3 activation pathway. British journal of pharmacology.

[13] Seitz CS, Lin Q, Deng H, Khavari PA (1998). Alterations in NF-κB function in transgenic epithelial tissue demonstrate a growth inhibitory role for NF-κB. Proceedings of the National Academy of Sciences.

[14] Sethi G, Tergaonkar V (2009). Potential pharmacological control of the NF-kappaB pathway. Trends in pharmacological sciences.

[15] Sung B, Kunnumakkara AB, Sethi G, Anand P, Guha S, Aggarwal BB (2009). Curcumin circumvents chemoresistance in vitro and potentiates the effect of thalidomide and bortezomib against human multiple myeloma in nude mice model. Mol Cancer Ther.

[16] Woo CC, Kumar AP, Sethi G, Tan KH (2012). Thymoquinone: potential cure for inflammatory disorders and cancer. Biochemical pharmacology.

[17] Sethi G, Ahn KS, Aggarwal BB (2008). Targeting Nuclear Factor-κB Activation Pathway by Thymoquinone: Role in Suppression of Antiapoptotic Gene Products and Enhancement of Apoptosis. Molecular Cancer Research.

[18] Woo CC, Hsu A, Kumar AP, Sethi G, Tan KH (2013). Thymoquinone Inhibits Tumor Growth and Induces Apoptosis in a Breast Cancer Xenograft Mouse Model: The Role of p38 MAPK and ROS. PloS one.

[19] Demchenko YN, Kuehl WM (2010). A critical role for the NFkB pathway in multiple myeloma. Oncotarget.

[20] Kannaiyan R, Hay HS, Rajendran P, Li F, Shanmugam MK, Vali S, Abbasi T, Kapoor S, Sharma A, Kumar AP, Chng WJ, Sethi G (2011). Celastrol inhibits proliferation and induces chemosensitization through down-regulation of NF-kappaB and STAT3 regulated gene products in multiple myeloma cells. Br J Pharmacol.

[21] Li Z-W, Chen H, Campbell RA, Bonavida B, Berenson JR (2008). NF-κB in the pathogenesis and treatment of multiple myeloma. Current Opinion in Hematology.

[22] Manu KA, Shanmugam MK, Ramachandran L, Li F, Fong CW, Kumar AP, Tan P, Sethi G (2012). First evidence that γ-tocotrienol inhibits the growth of human gastric cancer and chemosensitizes it to capecitabine in a xenograft mouse model through the modulation of NF-κB pathway. Clinical Cancer Research.

[23] van de Donk NW, Bloem AC, Lokhorst HM (2006). New treatment strategies for multiple myeloma by targeting BCL-2 and the mevalonate pathway. Current pharmaceutical design.

[24] Kannaiyan R, Manu KA, Chen L, Li F, Rajendran P, Subramaniam A, Lam P, Kumar AP, Sethi G (2011). Celastrol inhibits tumor cell proliferation and promotes apoptosis through the activation of c-Jun N-terminal kinase and suppression of PI3 K/Akt signaling pathways. Apoptosis : an international journal on programmed cell death.

[25] Agarwal A, Ghobrial IM (2012). Monoclonal Gammopathy of Undetermined Significance and Smoldering Multiple Myeloma: A Review of the Current Understanding of Epidemiology, Biology, Risk Stratification, and Management of Myeloma Precursor Disease. Clinical Cancer Research.

[26] Hideshima T, Podar K, Chauhan D, Anderson KC (2005). Cytokines and signal transduction. Best Practice & Research Clinical Haematology.

[27] Burger JA, Kipps TJ (2006). CXCR4: a key receptor in the crosstalk between tumor cells and their microenvironment. Blood.

[28] Möller C, Strömberg T, Juremalm M, Nilsson K, Nilsson G (2003). Expression and function of chemokine receptors in human multiple myeloma. Leukemia.

[29] Hideshima T, Chauhan D, Hayashi T, Podar K, Akiyama M, Gupta D, Richardson P, Munshi N, Anderson KC (2002). The Biological Sequelae of Stromal Cell-derived Factor-1α in Multiple Myeloma 1 This work was supported by Multiple Myeloma Research Foundation Senior Awards (to T. H. and D. C.), Leukemia and Lymphoma Society Scholar Award (to N. M.), NIH Grant PO-1 78378, and the Doris Duke Distinguished Clinical Research Scientist Award (to K. A.).1. Molecular Cancer Therapeutics.

[30] Rodríguez D, Morrison CJ, Overall CM (2010). Matrix metalloproteinases: what do they not do? New substrates and biological roles identified by murine models and proteomics. Biochimica et Biophysica Acta (BBA)-Molecular Cell Research.

[31] Zdzisińska B, Walter-Croneck A, Dmoszyńska A, Kandefer-Szerszeń M (2006). Matrix metalloproteinase and cytokine production by bone marrow adherent cells from multiple myeloma patients. Archivum immunologiae et therapiae experimentalis.

[32] Broek IV, Asosingh K, Allegaert V, Leleu X, Facon T, Vanderkerken K, Van Camp B, Van Riet I (2004). Bone marrow endothelial cells increase the invasiveness of human multiple myeloma cells through upregulation of MMP-9: evidence for a role of hepatocyte growth factor. Leukemia.

[33] Van Valckenborgh E, Croucher PI, De Raeve H, Carron C, De Leenheer E, Blacher S, Devy L, Noël A, De Bruyne E, Asosingh K (2004). Multifunctional role of matrix metalloproteinases in multiple myeloma: a study in the 5T2MM mouse model. The American journal of pathology.

[34] Demchenko YN, Glebov OK, Zingone A, Keats JJ, Bergsagel PL, Kuehl WM (2010). Classical and/or alternative NF-κB pathway activation in multiple myeloma. Blood.

[35] Berenson JR, Ma HM, Vescio R (2001). The role of nuclear factor-κB in the biology and treatment of multiple myeloma. Seminars in oncology: Elsevier.

[36] Baumann P, Mandl-Weber S, Oduncu F, Schmidmaier R (2008). Alkylating agents induce activation of NFκB in multiple myeloma cells. Leukemia research.

[37] Takahashi H, Shibuya M (2005). The vascular endothelial growth factor (VEGF)/VEGF receptor system and its role under physiological and pathological conditions. Clinical Science.

[38] Dvorak HF (2002). Vascular permeability factor/vascular endothelial growth factor: a critical cytokine in tumor angiogenesis and a potential target for diagnosis and therapy. Journal of clinical oncology.

[39] Aguayo A, Kantarjian H, Manshouri T, Gidel C, Estey E, Thomas D, Koller C, Estrov Z, O'Brien S, Keating M (2000). Angiogenesis in acute and chronic leukemias and myelodysplastic syndromes. Blood.

[40] Salven P, Orpana A, Teerenhovi L, Joensuu H (2000). Simultaneous elevation in the serum concentrations of the angiogenic growth factors VEGF and bFGF is an independent predictor of poor prognosis in non-Hodgkin lymphoma: a single-institution study of 200 patients. Blood.

[41] Bellamy WT (2001). Expression of vascular endothelial growth factor and its receptors in multiple myeloma and other hematopoietic malignancies. Seminars in oncology: Elsevier.

[42] Molica S, Vitelli G, Levato D, Gandolfo GM, Liso V (1999). Increased serum levels of vascular endothelial growth factor predict risk of progression in early B-cell chronic lymphocytic leukaemia. British journal of haematology.

[43] Feinman R, Koury J, Thames M, Barlogie B, Epstein J, Siegel DS (1999). Role of NF-κB in the rescue of multiple myeloma cells from glucocorticoid-induced apoptosis by bcl-2. Blood.

[44] Guo Y, Xu F, Lu T, Duan Z, Zhang Z (2012). Interleukin-6 signaling pathway in targeted therapy for cancer. Cancer treatment reviews.

[45] Uchiyama H, Barut BA, Mohrbacher AF, Chauhan D, Anderson KC (1993). Adhesion of human myeloma-derived cell lines to bone marrow stromal cells stimulates interleukin-6 secretion. Blood.

[46] Neufeld G, Cohen T, Gengrinovitch S, Poltorak Z (1999). Vascular endothelial growth factor (VEGF) and its receptors. The FASEB Journal.

[47] Jöhrer K, Janke K, Krugmann J, Fiegl M, Greil R (2004). Transendothelial migration of myeloma cells is increased by tumor necrosis factor (TNF)-α via TNF receptor 2 and autocrine up-regulation of MCP-1. Clinical cancer research.

[48] Bartlett JB, Dredge K, Dalgleish AG (2004). The evolution of thalidomide and its IMiD derivatives as anticancer agents. Nature Reviews Cancer.

